# International differences in the technical qualities of place of death classifications and data: A survey of researchers’ perspectives

**DOI:** 10.1177/02692163261468152

**Published:** 2026-08-03

**Authors:** Mayra Delalibera, Sílvia Lopes, Inês Dias da Silva, Barbara Gomes

**Affiliations:** 1 Faculty of Medicine, University of Coimbra, Portugal; 2 NOVA National School of Public Health, CHRC, REAL, CCAL, NOVA University Lisbon, Portugal; 3 Cicely Saunders Institute of Palliative Care, Policy and Rehabilitation, King’s College London, UK

**Keywords:** death certificates, classification, surveys and questionnaires, palliative care, public health

## Abstract

**Background:**

Place of death is a key population-level indicator for palliative care and health services planning. However, substantial international variation in how it is recorded limits cross-country comparisons and health system evaluations.

**Aim:**

Analyse the technical qualities of national place of death classifications and data, and identify strengths and weaknesses from the perspective of researchers using death certificate data.

**Design:**

Cross-sectional online survey. The questionnaire design was informed by the United Nations (UN) and World Health Organization (WHO) recommendations for international statistical and health classifications, and included closed- and open-ended questions.

**Setting/participants:**

Sixteen researchers identified through published place of death studies participated. Collectively, they had analysed data from 67 countries until 2022, most for over 10 years.

**Results:**

Most researchers reported that national classifications were stable, had mutually exclusive categories, and included a category for “home”. However, shortcomings were identified: lack of detailed categories for relevant settings (e.g. hospice, nursing home), ambiguous or inconsistent terminology, and limited use of hierarchical or multi-axial structures. Participants emphasised the need for greater standardisation of categories, clearer definitions, and improved data quality through training on completing death certificates.

**Conclusions:**

This is the first study to assess place of death classifications and data from researchers’ perspectives. Findings highlight critical limitations in current classification systems and provide guidance for developing an international classification aligned with UN and WHO recommendations. This will enable more meaningful cross-country comparisons and strengthen evidence to inform palliative care and health services planning worldwide.

**What is already known about the topic?**

- Place of death is a key indicator for palliative care and health system planning.
- National place of death classifications vary widely in categories and terminology.
- This variability hinders international comparison and monitoring.

**What this paper adds?**

- Existing place of death classifications from 67 countries are perceived as generally stable and having its own category for “home”.
- Place of death researchers identify lack of detail, ambiguous terms, and limited hierarchical structure as shortcomings of existing classifications.
- Important inconsistencies exist in the recording of place of death, particularly for nursing homes, hospices, and other long-term care settings.

**Implications for practice, theory or policy**

- The findings support the development of a standardised international place of death classification, with clearer improvements.
- Greater comparability and reliability of data would strengthen cross-country research.
- More robust and comparable place of death data can guide service planning and policy on palliative care worldwide.

## Background

Place of death is a key indicator for planning palliative care. It reflects healthcare accessibility, system performance, and alignment with people’s preferences.^1–3^ Discrepancies between preferred and actual place of death can indicate gaps in care provision and socioeconomic inequalities.^4^

Despite its importance, the way place of death is recorded and classified (e.g. in death certificates) varies between countries,^5^ ranging from broad categories (“home”, “hospital”, “other”) to more detailed systems including “nursing home”, “hospice”, or “emergency department”.^1,5,6^ This lack of standardisation hinders data comparability, limits the global understanding of trends in end-of-life care, and compromises quality monitoring.^3^

This study aimed to analyse the technical qualities of the place of death classifications used in different countries, focusing on features recommended by the United Nations (UN)^7^ and the World Health Organization (WHO)^8^ for statistical and health classifications. We also aimed to understand the perceived strengths and weaknesses of the existing classifications and data from the perspective of researchers using death certificate data.

## Methods

We conducted a cross-sectional, restricted-access online survey to explore researchers’ perspectives. The results were reported following the Checklist for Reporting of Survey Studies (CROSS).^9^

### Participants

Participants were researchers who had published observational, population-based studies reporting place of death as a primary or key outcome variable, using death certificate data in single- or multi-country settings. Studies were identified through database searches (MEDLINE, PsycINFO, EMBASE via Ovid, CINAHL, and Web of Science) from inception to November 2024.

Place of death had to be explicitly reported and analysed. Studies limited to a specific care setting (e.g. hospital-only) were excluded.

A total of 35 main authors (first or corresponding authors) were invited to participate; 26 from single-country studies and 9 from cross-national studies. First and corresponding authors were selected, as they are most likely to have in-depth knowledge of the data and the classification system used.

### Data collection

Guided by United Nations’ guidelines (UN) on the development of international statistical classifications^7^ and the technical qualities of health classifications defined by the World Health Organization (WHO)^8^ (details in Supplemental Appendix 1), we developed two questionnaire versions: one for authors of single-country studies and another for authors of multi-country studies. Both versions included closed- and open-ended questions assessing the technical properties of the classifications, including whether they are hierarchical or multi-axial (i.e. enabling aggregation of individual codes into broader categories; for example, having subcategories for places within hospital that can be combined into a single “hospital” category), exhaustive and mutually exclusive, stable (i.e. not changed too frequently or without proper review, justification, and documentation), inclusive of categories for key concepts (“home”, for example), unambiguously defined, and supported by a documented design process. Full versions of the questionnaires are provided in Supplemental Appendices 2 and 3 (single- and multi-country, respectively).

### Survey administration

Invitations to participate were sent by e-mail (August 2023–November 2024), with three reminders. The survey was created using LimeSurvey (Version 6.14.2) in English and all participants received a unique access code.

We received 16 completed questionnaires. Thirteen researchers did not respond to the invitation and six refused (four had not worked on this topic recently and two had retired).

The study is part of the EOLinPLACE Project^10^ approved by the Ethics Committee of the University of Coimbra (CE-068-2022) and registered in Research Registry (UIN 9213). All participants signed an informed consent.

### Data analysis

We used descriptive analysis to analyse the responses to the closed questions and thematic analysis principles according to Braun and Clarke^11^ to analyse themes and patterns in the open-ended questions.

## Results

The 16 researchers included had studied place of death data from 67 countries across all continents. Twelve researchers conducted single-country studies and four researchers analysed data from multi-country studies (Table 1).

**Table 1.** Characteristics of participants.

Participants (*n* = 16)	Authors of single-country studies: 12 participants (Brazil, Belgium, Czech Republic, Denmark, Finland, Germany, Italy, Portugal, Sweden, USA, and United Kingdom) Authors of multi-country studies: 4 participants (Australia, Belgium, Canada, and Germany)
Countries (*n* = 67)	Single-country studies (11 countries): Brazil Belgium (Flanders, Belgium and Brussels region) Czech Republic Denmark Finland (also 6 largest cities and rest of the country) Germany (selected regions and cities of North-Rhine Westphalia) Italy (Reggio Emilia province) Portugal Sweden USA United Kingdom (England, Wales, Scotland and Northern Ireland) Multi-country studies (63 countries): Argentina, Australia, Austria, Belgium, Botswana, Brazil, Canada, Chile, China, Colombia, Costa Rica, Croatia, Cuba, Cyprus, Czech Republic, Ecuador, Egypt, El Salvador, Estonia, France, Guatemala, Hungary, Iran, Iraq, Ireland, Italy, Japan, Kuwait, Latvia, Malaysia, Malta, Mexico, Mongolia, Mozambique, Nepal, New Zealand, Nicaragua, Norway, Panama, Papua New Guinea, Paraguay, Peru, Philippines, Spain (Andalusia), Singapore, South Africa, South Korea, Sri Lanka, Sweden, Thailand, the Netherlands, Trinidad and Tobago, Tunisia, United Kingdom (England, Wales, Scotland and Northern Ireland) Uruguay, USA, Specific region(s) in: Angola (1 demographic surveillance site), Burkina Faso (1 demographic surveillance site), Kenya (2 demographic surveillance sites), Tanzania (1 demographic surveillance site), Ethiopia (2 demographic surveillance sites), Zambia (4 demographic surveillance sites), Bangladesh (1 demographic surveillance site)^a^
Last year of used data	Single-country studies: 2021 Multi-country studies: 2022
Number of years using data (n)	
Up to 5 years	2
6–10 years	3
11–15 years	5
16–20 years	3
More than 20 years	3
Research team excluding the participant (n)	
Up to 5 researchers	8
6–10 researchers	4
11–15 researchers	2
More than 20 researchers	2

a Reported exactly as answered by the researcher in the survey.

The most recent year of place of death data used by researchers was 2022, and most had been using data for more than 10 years. Half worked in teams of ⩾6 researchers (Table 1).

In most countries, place of death was reported by the doctor/physician who completed the death certificate (Supplemental Table S1). In most countries, national health or statistical institutions were responsible for providing and classifying the data.

The most frequent groups of categories of place of death analysed were “hospital or health institution” (15 researchers), “home” (14 researchers), “other defined” (10 researchers) and “ill-defined” (10 researchers; Supplemental Table S2).

**Figure 1.**
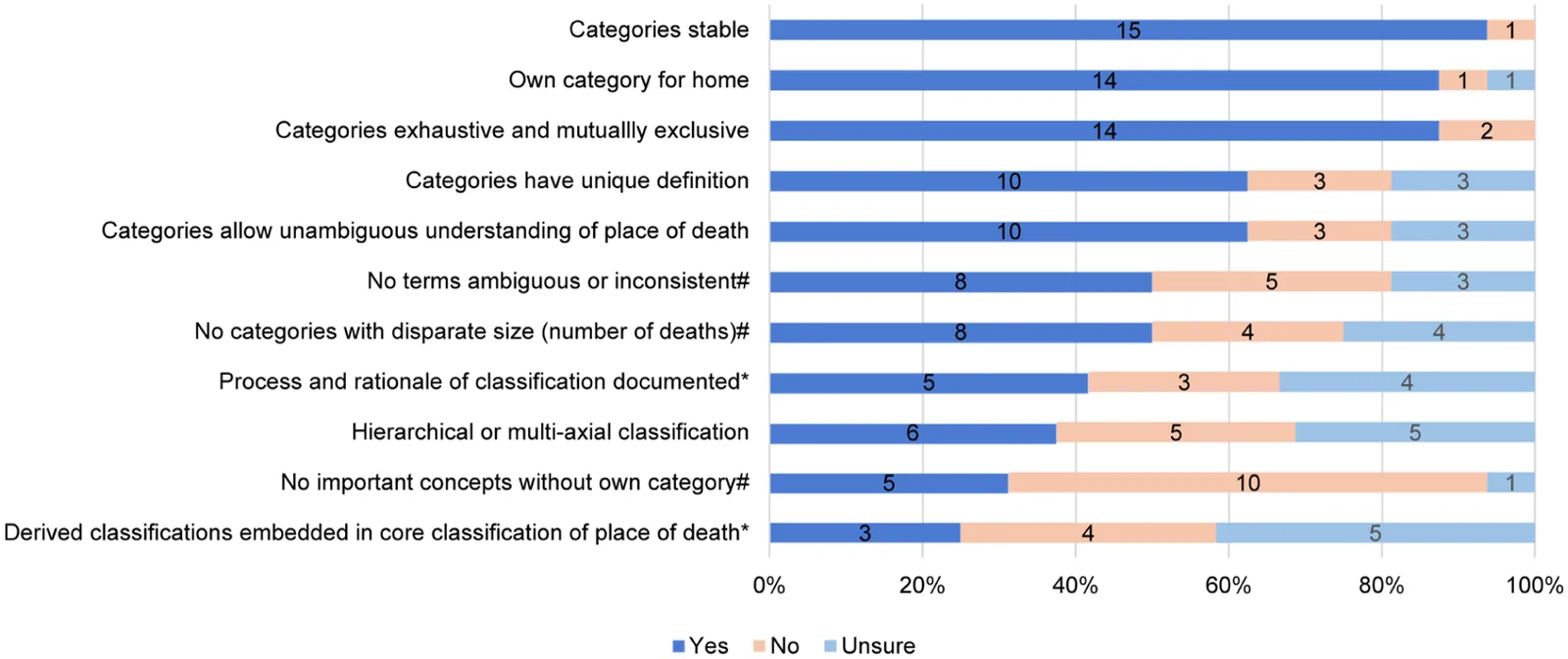
Researchers’ appraisal of the technical qualities of existing place of death classifications. *These questions were not included in the survey of researchers working with multi-country data. #Answers to these questions were inverted for easier interpretation.

Regarding the technical qualities of existing place of death classifications used by researchers in their studies, almost all researchers considered the classifications were stable, exhaustive and mutually exclusive, with “home” having its own category (Figure 1). However, several researchers identified shortcomings. Ten noted the lack of categories for important concepts such as nursing homes, assisted living facilities, mental hospitals and prisons. Five mentioned ambiguous or inconsistent terms (e.g., other healthcare facility, home, and nursing home) and four indicated there were categories with disparate population size. Only 6 of the 16 researchers reported that it was possible to aggregate data from individual codes into broader categories (i.e. classification was hierarchical or multi-axial; Figure 1). Only 5 of the 16 researchers reported that the process and rationale for designing the classification was documented (Figure 1).

Analysing the open-ended answers we found that the existing place of death classifications did not comply with all UN and WHO recommendations, with unclear or overlapping terms and limited number of categories, as explained by Participant 14: *“In Hungary, the death certificate only contains two categories of place of death, hospital and others, whereas hospice (e.g., palliative care institution) was only available as a category in England, Wales, New Zealand, Canada, and the USA. In Mexico, nursing home/care home is not a recorded category”*. In other countries, key terms do not even exist in the native language, as referred to by Participant 9: “*The location ‘nursing home’ is not easily translated into Swedish, since the organisation type/institution of nursing homes is supposed to no longer exist in our services for old people in particular*.” Some places do not have their own category, as mentioned by Participant 11: *“Mental hospitals, prisons, sports pitch, schools, etc. cannot be identified”*, or have an ambiguous meaning, as referred by Participant 1: *“When considering other healthcare facility, it is not possible to differentiate long-term home care/nursing homes/hospices from emergency care services.”* Participant 10 also highlighted that it is *“Interesting to consider ambiguous meaning of home – i.e. home includes another person’s home, which may not be home to that person. Also, care or nursing homes may represent home to some decedents but not to others.”*

Three themes emerged from the researchers’ suggestions for improvements:

1. Need for standardisation of categories: Participants, like Participant 3, emphasised the importance of having comparable categories of place of death across countries: *“[We need] An international classification that will allow for comparable data.”* Participants also highlighted the need to standardise place of death categories in death certificates, through clear definitions and distinctions to reduce ambiguity. Participant 14 wrote: *“[I suggest] Minimal distinction of home, hospital, care home/nursing home, palliative care setting, other. . . possibly distinguish different types of hospital wards (ER, ICU are very different than geriatrics ward).”* Participant 5 called for the classification of place of death to be a uniform and official document *“The place of death would have to be uniformly documented as tickable categories in the federal death certificates [such as]: a) home; b) hospital: b1) intensive care unit, b2) palliative care unit, b3) other wards, hospital locations; c) inpatient hospice; d) nursing home e) other places.”*
2. Need to improve data quality: Participants highlighted the lack of reliable data and the need for guidelines and training to ensure consistent and accurate completion of death certificates. In Participant 6′s words: *“We need instruction to make more reliable the classification”* and Participant 12 called for a “*Universal methodology of coding similar to causes of death (IRIS etc.).”* Participant 11 emphasised the need to *“Develop recommendations for the governments and agencies in Europe and beyond that are responsible for the content of the death certificate*.” Participant 15 summarised: *“[It is necessary] Homogenity of categories and a good dictionary. Training for people that have to write the death certificates. Increase awareness of the importance of this document.”*
3. Difficulties in accessing data: Participants noted the importance of ensuring that death certificates are completed correctly and in full, as suggested by Participant 13: “*Ensure it is always completed (*i.e. *no missing values) and made publically available, either through unit record data or in published reports.”* This participant also stated that “*Part of the reason [for the difficulty in accessing data] is incomplete registration of deaths, while another reason is that it is not considered a priority indicator, even though it should be straightforward to record this.”* Participant 15 mentioned “*The high amount of missing values in some countries. The possibility of nonregistered deaths* *for example* *in the rural area, forest or even slums.”*

## Discussion

### Main findings

This study advances research on place of death by assessing the technical qualities of national place of death classifications through the perspectives of researchers using death certificate data. While many existing classifications are viewed as stable, with mutually exclusive categories, including a category for “home”, significant limitations persist, such as lack of detailed categories, ambiguous terminology, and limited hierarchical structure. Studies indicate considerable variation in how countries record and categorise place of death.^1,5^ Some countries and death certificates detail the places and some list only “hospital”, “home”, or “other”.^6^ This lack of specificity obscures crucial end-of-life care settings, such as hospices and palliative care facilities, and hinders national and international comparisons, with implications for monitoring healthcare system performance and patient experiences.

### What this study adds

As referred to by the researchers surveyed, most national classifications lack hierarchical or multi-axial structures. This is a critical shortcoming, as hierarchical classifications enable a flexible use of data at different levels of specificity, allowing data grouping.^10^ Researchers also identified ambiguity in how terms such as “home”, “health unit”, and “nursing home” are defined and classified in different countries.

The study also confirms concerns about the lack of documentation on classification development, poor quality or incomplete death certification systems, particularly for community deaths in less developed countries, as noted by Adair.^12^ These deficiencies call for harmonisation and reinforce the need for a clear, well-documented global standard.

An international place of death classification is timely needed, as proposed by Namukwaya et al.^10^ Such a classification should be clear, exhaustive, with mutually exclusive categories, adaptable to health systems at a global level, including countries in low-resource settings.^12^ Training professionals who complete death certificates is essential, as incorrect completion compromises data quality and weakens policy and planning.

These findings also have methodological implications for researchers using place of death data. We recommend that researchers systematically document and report the technical qualities of the classification system used in their studies. They should also acknowledge any limitations arising from non-hierarchical or undocumented systems when interpreting and comparing results across countries, and engage with data providers and health authorities to advocate for clearer, more standardised classifications. The study included researchers who have worked with data across countries at different income levels; thus, despite the underrepresentation of lower-income settings, it provides a perspective on the technical qualities of place of death classifications that is not restricted to high-income countries.

### Strengths and limitations of the study

A strength of this study is the inclusion of researchers with experience analysing place of death data from more than 60 countries across all continents, using a framework informed by UN and WHO recommendations for health classifications. However, the study has limitations such as a small sample size, limiting statistical analysis. The survey was in English, and the responses reflect individual researchers’ interpretations, which may not fully capture all administrative or policy factors that may influence classification systems. Finally, the lack of obligation to respond to some open-ended questions limited the amount of data collected. Additionally, although the survey included authors of studies from all continents, certain regions (e.g. Sub-Saharan Africa and South/Southeast Asia) may be underrepresented, which should be considered when generalising the findings to a global context.

Despite these limitations, this study provided critical insights that supported the development of an international classification by our research team. In March 2026, this new classification, called “International Classification of Dying Places” (ICP),^13^ has been endorsed by the United Nations Statistical Commission as an international classification, aimed to strengthen cross-country comparisons and improve end-of-life care monitoring worldwide.

## Conclusion

Researchers using death certificate data from over 60 countries recognise both the strengths and limitations of current national place of death classifications. While many are stable and have mutually exclusive categories, areas for improvement were identified such as creating more specific and unambiguous categories, and standardising classifications across countries, with a view to harmonising place of death information globally.

The findings directly informed the development of the pioneering International Classification of Dying Places (ICP) which will allow more meaningful data and comparisons across and within countries, informing service planning and improving palliative care.

## Supplemental Material

sj-docx-1-pmj-10.1177_02692163261468152 – Supplemental material for International differences in the technical qualities of place of death classifications and data: A survey of researchers’ perspectives Supplemental material, sj-docx-1-pmj-10.1177_02692163261468152 for International differences in the technical qualities of place of death classifications and data: A survey of researchers’ perspectives by Mayra Delalibera, Sílvia Lopes, Inês Dias da Silva and Barbara Gomes in Palliative Medicine

## Data sharing statement

Anonymised data that support the findings of this study may be available from the corresponding author (BG) upon reasonable request.*
