# Supplementary material for: International differences in the technical qualities of place of death classifications and data: A survey of researchers’ perspectives
Source: Palliat Med. 2026 Aug 3;40(9):1420–6. doi: 10.1177/02692163261468152 (PMC13612875; doi:10.1177/02692163261468152)
Supplement: Supplementary file 1 [file sj-docx-1-pmj-10.1177_02692163261468152.docx]

**I**nternational differences in the technical qualities of place of death classifications and data: a survey of researchers’ perspectives

**Supplementary material**

**Appendix A**

Technical qualities for health classifications defined by the World Health Organization (WHO)^1^ and recommendations by the United Nations (UN)^2^ for international statistical classifications

| According to WHO guidelines, the following technical qualities should be demonstrated to enable a decision to be made on the inclusion of a classification in the WHO Family of International Classifications: | According to best practice guidelines authored by the Chair of the UN Expert Group on International Statistical Classifications, the essential components of an statistical classification are: |
| --- | --- |
| - Each classification should have a hierarchical or multi-axial structure such that it is possible to aggregate data from individual codes into broader categories.  - Classification categories should be exhaustive and mutually exclusive.  - The categories are stable, i.e. they are not changed too frequently or without proper review, justification and documentation.  - A concept within a classification that is of importance should have its own category.  - Categories within a classification should facilitate the description of phenomena in a way that allows unambiguous understanding by others, including statistical users.  - Each category or concept should have a unique definition.  - Terms should not be ambiguous, and the relationship between terms should be consistent.  - The classification should not have categories at the same level in its hierarchy that are too disparate in their population size, i.e. there should be consistent granularity.  - Derived classifications are always embedded in the core classification. | - A consistent conceptual basis;  - A flat or hierarchic structure;  - Categories that are mutually exclusive and exhaustive;  - Definitions that are clear and unambiguous, and which define the content of each category;  - That it is up-to-date and relevant;  - That it is robust enough to last for a period of time;  - That it meets user needs;  - That it provides comparability over time and between collections;  - That it provides guidelines for coding and output of data collected using it. |

1. WHO. WHO Family of International Classifications (WHO-FIC): Development Guidelines. Geneva: WHO; 2021.
2. UN. Best Practice Guidelines for Developing International Statistical Classifications: UN; 2013. <https://unstats.un.org/unsd/classifications/bestpractices>

**Appendix B**

**EOLinPLACE International survey of key researchers who have worked on single-country studies about place of death**

Please answer the following survey questions reporting to your most recent use of place of death data from the country you live and how place of death was classified in death certificates by then.

1. Specify the most recent year of place of death data you used from the country you live in:

**2. Please provide us some basic information about the data and the classification:**

2.1. Custodians (organizations providing the data and responsible for the classification, please specify both if they differ):

2.2. Geographical remit (name of country or regions if not whole country examined):

2.3. Informant on place of death (e.g., bereaved relative, doctor certifying the death):

2.4. Categories of latest version you used (name all in the original language and in English if possible, including residual/ill-defined categories, higher and lower level categories when available)

2.5. Classification changes since 2012 (if yes, specify which and indicate years of revisions)

2.6. Link to blank death certificate in the most recent year of data used, if available (or send by email).

2.7. Copies of blank death certificate/registration in the most recent year of data used, if possible (send by email).

2.8. Links or copies (send by email) to supporting documentation, including instructions on how to classify place of death and statistical/scientific reports of place of death data, if available.

2.9. Additional information on place of death classification and data.

**3. Please respond to each of the following questions, explaining each answer in the box. Please refer to your most recent use of place of death data in the country you live in.**

3.1. Is the classification hierarchical or multi-axial such that it is possible to aggregate data from individual codes into broader categories?

_____Yes ____No _____Unsure

**Please, justify your answer:**

3.2. Are the categories of place of death exhaustive and mutually exclusive?

_____Yes ____No _____Unsure

**Please, justify your answer:**

3.3. Are the categories of place of death stable? This means they are not changed too frequently or without proper review, justification and documentation.

_____Yes ____No _____Unsure

**Please, justify your answer:**

3.4. Does the concept of “home” have its own category?

_____Yes ____No _____Unsure

**Please, justify your answer:**

3.5. Are there any other concepts of importance in your country that do not have their own category?

_____Yes ____No _____Unsure

**Please, justify your answer:**

3.6. Do the categories facilitate the description of the phenomenon “place of death” in a way that allows unambiguous understanding by others, including statistical users?

_____Yes ____No _____Unsure

**Please, justify your answer:**

3.7. Does each category or concept of place of death have a unique definition?

_____Yes ____No _____Unsure

**Please, justify your answer:**

3.8. Are any of the terms ambiguous or is any relationship between terms inconsistent?

_____Yes ____No _____Unsure

**Please, justify your answer:**

3.9. Does the classification have categories at the same level in the hierarchy that are too disparate in population size (i.e., number of deaths)?

_____Yes ____No _____Unsure

**Please, justify your answer:**

3.10. Are there any other classifications that derive from the classification of place of death, and if yes are they embedded in the core classification?

_____Yes ____No _____Unsure

**Please, justify your answer:**

3.11. Has the process and rationale for designing the classification been documented?

_____Yes ____No _____Unsure

**Please, justify your answer:**

**4. Final questions:**

4.1. Since when you have been using place of death data from the country you live in:

___up to 5 years,

___6-10 years,

___11-15 years,

___16-20 years,

___more than 20 years

4.2. How many other researchers from your team (apart from you) have used the data:

___ up to 5 researchers,

___6-10 researchers,

___11-15 researchers,

___16-20 researchers,

___more than 20 researchers

4.3. Any suggestions to improve the place of death classification and data in the country you live in:

4.4. Any suggestions to inform the development of an international classification:

4.5. Any suggestions to improve the quality of place of death data internationally:

**Thank you for your participation in our survey!**

We will contact you only if any clarification of your responses is required and to send a brief summary of results, if you indicated that preference.

**Appendix C**

**EOLinPLACE International survey of key researchers who have worked on multi-country studies about place of death**

Please answer the following questions reporting to your latest multi-country study on place of death data and how place of death was classified in death certificates by then.

**1. Please provide us some basic information about the data and the classification.**

1.1. Geographical remit (name of countries or regions if not whole country examined):

1.2 Temporal remit (range of years examined):

1.3. Categories of place of death which were the focus of analysis across countries and why:

**2. Please respond to each of the following questions, explaining each answer in the box. Please refer to your most recent use of place of death data in the countries you studied.**

2.1. Were the classifications of place of death from the different countries hierarchical or multi-axial such that it is possible to aggregate data from individual codes into broader categories?

_____Yes, for all countries

____Yes, but not all. Which countries had hierarchical or multi-axial classifications?

____No

_____Unsure

**Please, justify your answer:**

2.2. Were the categories of place of death exhaustive and mutually exclusive in the countries you studied? If not, in how many?

_____Yes, for all countries

____Yes, but not all. Which countries had exhaustive and mutually exclusive categories? ____No

_____Unsure

**Please, justify your answer:**

2.3. Were the categories of place of death stable in the countries you studied? This means they were not changed too frequently or without proper review, justification and documentation.

_____Yes, for all countries

____Yes, but not all. Which countries had stable categories?

____No

_____Unsure

**Please, justify your answer:**

2.4. Did the concept of “home” have its own category in the countries you studied?

_____Yes, for all countries

____Yes, but not all. In which countries did “home” had its own category?

___No

_____Unsure

**Please, justify your answer:**

2.5. Were there any other concepts of importance that did not have their own category in the countries you studied?

_____Yes, for all countries

____Yes, but not all. Which important concepts did not have their own category and in which countries?

____No

_____Unsure

**Please, justify your answer:**

2.6. Did the categories facilitate the description of the phenomenon “place of death” in a way that allowed unambiguous understanding by others, including statistical users?

_____Yes, for all countries

____Yes, but not all. In which countries did the categories allowed unambiguous understanding by others?

____No

_____Unsure

**Please, justify your answer:**

2.7. Did each category or concept of place of death have a unique definition?

_____Yes, for all countries

____Yes, but not all. Which countries had a unique definition for each category or concept? ____No

_____Unsure

**Please, justify your answer:**

2.8. Were any of the terms ambiguous or were any relationship between terms inconsistent?

_____Yes, for all countries

____Yes, but not all. Which countries did have ambiguous or inconsistent relationship between terms?

____No

_____Unsure

**Please, justify your answer:**

2.9. Did the classifications have categories at the same level in the hierarchy that were too disparate in population size (i.e., number of deaths)?

_____Yes, for all countries

____Yes, but not all. Which countries had categories at the same level that were too disparate in population size?

____No

**Please, justify your answer:**

**3. Final questions:**

3.1. Since when have you been using place of death data?

___up to 5 years,

___6-10 years,

___11-15 years,

___16-20 years,

___more than 20 years

3.2. How many other researchers from your team (apart from you) have used place of death data?

___ up to 5 researchers,

___6-10 researchers,

___11-15 researchers,

___16-20 researchers,

___more than 20 researchers

3.3. What were the main barriers you felt when conducting your study?

3.4. What are the least and most difficult categories to compare between countries?

3.5. In your opinion, which country(ies) was/were good example(s) of classification? If possible, please list the classification categories.

3.6. Any suggestions to improve the place of death classification and data in the countries you studied:

3.7. Any suggestions to inform the development of an international classification:

3.8. Any suggestions to improve the quality of place of death data internationally:

Please let us know if you wish to receive a brief summary of the findings by email.

___Yes ___No

**Thank you for your participation in our survey**!

We will contact you only if any clarification of your responses is required and to send a brief summary of results, if you indicated that preference.

Table S1 – Information about death certificate data

| Informant on place of death | **Doctor or physician certifying the death** - 7 countries  - Doctor certifying the death with a standardized form to the whole country  - Doctor certifying death  - Physician who carried out the post-mortem examination and filled out the death certificate himself/herself  - Doctor  - Doctor certifying the death  - Original data provided by a physician  - Doctor certifying the death  **Next of kin** - 1 country  - For US death certificate is based on next of kin report  **Unspecified -** 4 countries  - Information was based on the care days registered in the registries. We as researchers have derived the place of death based on this information  - We have not looked at this information  - Death certificates  - Death certificate |
| --- | --- |
| Custodians (organizations providing the data and responsible for the classification) | Mortality Information System - Ministry of Health (Brazil)  Finnish Institute for Health and Welfare (Finland)  National Institute of Statistics (Portugal)  Department of Epidemiology, AUSL IRCCS of Reggio Emilia (Italy)  Statistics Denmark  National Records of Scotland  National Board of Health and Welfare in Sweden (NBHW), Statistics Sweden, and the Swedish Register for Palliative Care (SRPC)  Office for National Statistics (ONS) England and Wales / Scottish National Safe Haven and eDRIS Team (Public Health Scotland) / Honest Broker Service (HBS) within the Business Place of death also available from NHS England  Regional Ministries of Public Health (death certificates are a regional responsibility in Belgium and we have been using those from Flanders and from the Brussels capital region).  Institute of Health Information and Statistics of the Czech Republic  We used the residential history file (USA)  Principal investigator (Germany) |
| Classification changes since 2012 | No: 11  Yes: 1 Brazil (in 2013, the place "Indigenous territory" was included, but it is rarely used in practice) |

Notes: These questions were not asked to the researchers who used multi-country data.

Free-text answers reported exactly as answered by the researcher in the survey.

Table S2 – Categories of place of death mentioned by the researchers

| **Home** | **Hospital or health institution** | **Other defined** | **Ill defined** |
| --- | --- | --- | --- |
| Home | Hospital | Public place | Unknown |
| Own residence | Other healthcare facility | Indigenous territory | Other |
| At home | Health center: primary care hospital | Long-term care facility | Other place |
|  | Emergency room (ER) | Nursing home | Elsewhere |
|  | Healthcare facility for inpatient care | Inpatient hospice unit | Don’t know/ |
|  | Other healthcare facility (ambulant care etc.) | Community (that includes Assisted Living Facilities and Homes) | Not available |
|  |  | Palliative care unit |  |
|  |  | Care home |  |
|  |  | Hospice |  |
|  |  | Public route |  |
|  |  | At work |  |
|  |  | On street or other public place |  |
|  |  | During transport to healthcare facility (ambulance car or any other vehicle) |  |
|  |  | In social care facility (elderly home etc.) |  |

Notes: the categories were reported exactly as written by the researcher in the survey, and we grouped them into the four large groups described in the first row of the table above.
